# Facing up to the hard question of consciousness

**DOI:** 10.1098/rstb.2017.0342

**Published:** 2018-07-30

**Authors:** Daniel C. Dennett

**Affiliations:** Center for Cognitive Studies, Tufts University, Medford, MA 02155, USA

**Keywords:** consciousness, Chalmers, block, virtual machines, degrees of freedom, access

## Abstract

The so-called hard problem of consciousness is a chimera, a distraction from the hard question of consciousness, which is once some content reaches consciousness, ‘then what happens?’. This question is seldom properly asked, for reasons good and bad, but when asked it opens up avenues of research that promise to dissolve the hard problem and secure a scientifically sound theory of how the human brain produces the (sometimes illusory) convictions that mislead us.

This article is part of the theme issue ‘Perceptual consciousness and cognitive access'.

## 1. The hard question is not the hard problem

David Chalmers (‘Facing up to the hard problem of consciousness’ [1]) focused the attention of people researching consciousness by drawing a distinction between the ‘easy’ problems of consciousness, and what he memorably dubbed the hard problem.

The easy problems of consciousness include those of explaining the following phenomena:

— the ability to discriminate, categorize and react to environmental stimuli;
— the integration of information by a cognitive system;
— the reportability of mental states;
— the ability of a system to access its own internal states;
— the focus of attention;
— the deliberate control of behaviour;
— the difference between wakefulness and sleep [1, p. 201].

The hard problem ‘is the problem of *experience*’ [1, p. 202], accounting for ‘what it is like’ [2] or *qualia.* To many researchers, this seemed like a sensible divide-and-conquer research strategy: first, we tackle all the easy problems, and then we turn our attention to the hard problem. To others, the identification of the hard problem called for something like a scientific revolution, replacing the standard methods and assumptions of cognitive science (which are continuous with the standard methods and assumptions of biology, chemistry and physics) with a more radical perspective. The hard problem, they surmised, will only be addressed by a return to some form of dualism, or panpsychism, or some yet to be articulated overthrow of what might be considered normal science.

I have been arguing for decades [3–8] that Chalmers was *mis-*focusing our attention, exaggerating an artefact of inquiry that I identified in 1991: the failure to ask, and answer, what I called the hard question: ‘And then what happens?’ [9, p. 255]. The question, more specifically, is: *once some item or content ‘enters consciousness’, what does this cause or enable or modify?* For several reasons, researchers have typically either postponed addressing this question or failed to recognize—and assert—that their research on the ‘easy problems’ can be seen as addressing and resolving aspects of the hard question, thereby indirectly dismantling the hard problem piece by piece, without need of any revolution in science. The causes of the misdirection can be uncovered by reminding ourselves of a few largely uncontentious but easily neglected discoveries of neuroscience:

(i) There is no double transduction [5]. The various peripheral and internal transducers—rods and cones, hair cells, olfactory epithelium cells, stretch-detectors in muscles, temperature-change detectors, nociceptors and others—are designed by evolution to take the occurrence of physically detectable properties as input and yield signals—axonal spike trains—as output. There is no central arena or depot where these spike trains become recipes for a second transduction that restores the properties transduced at the periphery, or translates them into some sort of counterpart properties of a privileged medium. Vision is not television, audition does not strike up the little band in the brain, olfactory perception does not waft aromas in any inner chamber. (Nor, one had better add, are there subjective counterpart properties, subjective colours-that-are-not-seen-with-eyes, inaudible-sounds, ghost-aromas that need no molecular vehicles, for us to enjoy and identify in some intimate but unimaginable way.) Colour vision is accomplished by a sophisticated system of information processing conducted entirely in spike trains, where colours are ‘represented’ by physical patterns of differences in spike trains that are not themselves colours. The key difference between the transmission of colour information by a DVD and the transmission of colour information by the various cortical regions is that the former is designed by engineers to be a recipe for *recreating* (via a transduction to another medium) the very properties that triggered the peripheral transducers that compose the megapixel screens behind the camera lens, while the latter is designed by evolution to deliver useful information about the affordances that matter to the organism in a form that is readily usable or consumable [10] by the specialized circuits that modulate the behaviours of systems external and internal.
(ii) So, there is no place in the system for qualia, *if they are conceived of as intrinsic properties instantiated by (as contrasted with represented by)* some activities in the nervous system.

I have discovered that it is useful to pause at this point and invite readers to consider whether or not they actually agree with these two basic points, because their implications are highly destructive of commonplace presumptions. In particular, the widespread conviction that qualia, thus conceived, *must obviously exist* if we are to make sense of our introspective access to them, is an illusion, not an optical illusion or auditory illusion, but a theorist's illusion, an artefact of bad theory, not observation. Richard Power nicely captures the source of this illusion.

We know that our perceptions or imaginings of trees, faces, etc. are distinct from the objects themselves. They are internal representations, representations in our minds.

We understand the concept of representation from external representations, such as pictures, or verbal descriptions. For these representations we can have direct experience of both a representer (e.g. portrait painting) and a representee (e.g. the person painted). Call these the medium and the content. Thus for the Mona Lisa, the medium is a painting that hangs in the Louvre; the content is an Italian woman who modelled for the artist centuries ago. Medium and content may have attributes in common, if the representation is iconic (as they say in Semiotics). Oval partly-brown patches in the painting resemble the oval brown eyes of the Italian lady. But usually medium and content are of different stuff: oil on canvas, in the case of the Mona Lisa, as against human flesh. And in many cases the representation is symbolic, so that medium and content share no features.

This is the conceptual scheme that we bring to internal representations, because it is the only one we have. But there is a huge difference. For external representations we can experience both medium and content, oil on canvas as well as people, trees, or whatever. But for internal representations, we do not experience the medium AT ALL. Only the content, along with some contextual features such as the time when the percept or imagining occurred. The idea of a spiritual consciousness arises from the illusion that we DO experience the medium of our internal representations, and that it is iconic.

…In short, we conceptualise the medium of our internal representations by abstracting some features from the content, and attributing them to some kind of spiritual or ghostly substance. That is the best we can do, because actually we cannot experience the medium at all and have to look for analogies in the external world. The idea that the medium is some state of the brain seems intuitively absurd, so powerful is the illusion that we are dealing with an iconic representation in a medium of spirit.

Personal correspondence, 3 May 2017

(iii) Consciousness cannot be a ‘movie running in the head’. All the work (and play) done by the homunculus who sits in the Cartesian Theater [9], the imaginary control room in the centre of the brain, must be broken up into tasks that can be outsourced to lesser agents or agencies. All the comprehension, appreciation, delight, revulsion, recognition, amusement, etc. that human beings experience must be *somehow* composed by the activities of billions of neurons that are myopic in the extreme, cloistered in their networks of interacting brethren, oblivious to the larger perspective they are helping to create. But how? That is the hard question.

## 2. Why the hard question is seldom asked

One explanation for the neglect of the hard question is that science in this area proceeds from the peripheries towards the interior, analysing the operation of transducers and following their effects inwards. Start with the low hanging fruit; it is a matter of proximity, non-invasiveness and *more reliable manipulability—*we can measure and control the stimulation of the peripheral transducers with great precision. Research on the efferent periphery, the innervation of muscles and the organization of higher-level neural motor structures, can be done, but is more difficult for a related reason, which has more general implications: controlled experiments are designed to isolate, to the extent possible, one or a few of the variable sources of input to the phenomenon—clamping the system, in short—and then measuring the dependent variables of output. Accomplishing this requires either invasive techniques (e.g. stimulation *in vivo* of motor areas) or indirect manipulation of subjects' motivation (e.g. ‘press button A when you hear the tone; press button B when you hear the click; try not to move otherwise’). In the latter case, researchers just assume, plausibly, that conscious subjects will understand the briefing, and be motivated to cooperate, and avoid interfering activities, mental or skeletal, with the result that they will assist the researcher in setting up a transient *virtual machine* in the cortex that restricts input to their motor systems to quite specific commands.

Similarly, working on the afferent side of the mountain, researchers brief subjects to attend to specific aspects of their sensory manifold, and to perform readily understood simple tasks (usually, as quickly as possible), with many repetitions and variations, all counterbalanced and timed. The result, on both the afferent and efferent fronts, is that subjects are systematically constrained—for the sake of science—to a tiny subset of the things they *can* do with their consciousness. Contrast this with non-scientific investigation of consciousness: ‘A penny for your thoughts’, ‘What are you looking at now?’, ‘What's up?’

This is all obvious, but it has a non-obvious side effect on the science of consciousness: it deflects attention from what is perhaps the most wonderful feature of human consciousness: the *general* answer to the hard question, ‘And then what happens?’ is ‘*Almost anything can happen!’* Our conscious minds are amazingly free-wheeling, open-ended, protean, untrammelled, unconstrained, variable, unpredictable, … . Omni-representational. Not only *can* we think about anything that *can* occur to us, and not only *can* almost anything (anything ‘imaginable’, anything ‘conceivable’) occur to us, but once something has occurred to us, we can respond to it in an apparently unlimited variety of ways, and then respond to those responses in another Vast [11, p. 109] variety of ways, and so forth, an expanding set of possibilities that outruns even the productivity of natural languages (words fail me). Of course, on any particular occasion, the momentary states of the various component neural systems constrain the ‘adjacent possible’ [12] to a limited selection of ‘nearby’ contents, but this changes from moment to moment, and is not directly in anybody's control. It is this background of omnipotentiality that we take for granted, and cordon off accordingly in our experimental explorations. It is worth noting that we have scant reason to think that simpler nervous systems have a similar productivity. Most are likely to be ‘cognitively closed’ [13] systems, lacking the representational wherewithal to imagine a century or a continent, or poetry, or democracy, … or God. The famous four Fs (feed, fight, flee and mate) may, with a few supplements (e.g. explore, sleep) and minor suboptions, exhaust the d*egrees of freedom* of invertebrates.

Probably even our closest relatives, the chimpanzees and bonobos, have severely constricted repertoires of representation, compared with us. Here is a simple example: close your eyes right now and imagine, in whatever detail you like, putting a plastic wastebasket over your head and climbing hand-over-hand up a stout rope. Easy? Not difficult, even if you are not strong and agile enough—most of us are not—to actually perform the stunt yourself. Could a chimpanzee engage in the same ‘imagining’ or ‘mental time travel’ or ‘daydreaming’? I chose the action and the furnishings to be items deeply familiar to a captive chimp; there is no doubt such a chimp could recognize, manipulate, play with the basket, and swing or climb up the rope, but does its mind have the sort of self-manipulability to put together these familiar elements in novel ways? Maybe, but maybe not. The abilities of clever animals—primates, corvids, cephalopods, cetaceans—to come up with inventive solutions to problems have been vigorously studied recently (e.g. [14–16]), and this research sometimes suggests that they are capable of trying out their solutions ‘off line’ in their imaginations before venturing them in the cruel world, but we should not jump to the conclusion that their combinatorial freedom is as wide open as ours. For every ‘romantic’ finding, there are ‘killjoy’ findings [17] in which clever species prove to be (apparently) quite stupid in the face of not so difficult challenges.

One of the recurrent difficulties of research in this area is that in order to conduct proper, controlled scientific experiments, the researchers typically have to impose severe restrictions on their animals' freedom of movement and exploration, and also submit them to regimes of training that may involve hundreds or even thousands of repetitions in order to ensure that they attend to the right stimuli at the right time and are motivated to respond in the right manner (the manner intended by the researcher). Human subjects, by contrast, can be uniformly briefed (in a language they all understand) and given a few practice trials, and then be reliably motivated to perform as requested for quite long periods of time [18]. The tasks are as simple as possible, in order to be accurately measured, and the interference of ‘mind-wandering’ can be minimized by suitable motivations, intervals of relaxation, etc.

The effect, in both speaking human subjects and languageless animal subjects, is to minimize the degrees of freedom that are being exploited by the subjects, in order to get clean data. So, huge differences in the available degrees of freedom are systematically screened off, neither measured nor investigated.

This explains the relative paucity of empirical research on language *production* in contrast with language *perception*, on *speaking* in contrast with *perceiving, parsing, comprehending.* What are the inputs to a controlled experiment on speaking? It is easy to induce subjects to read passages aloud, of course, or answer ‘Yes’ and ‘No’ to questions displayed, but if the experimenter were to pose a task along the lines of ‘tell me something of interest about your life’ or ‘what do you think of Thai cuisine?’ or ‘say something funny’, the channel of possible responses is hopelessly broad for experimental purposes.

**Figure 1.**
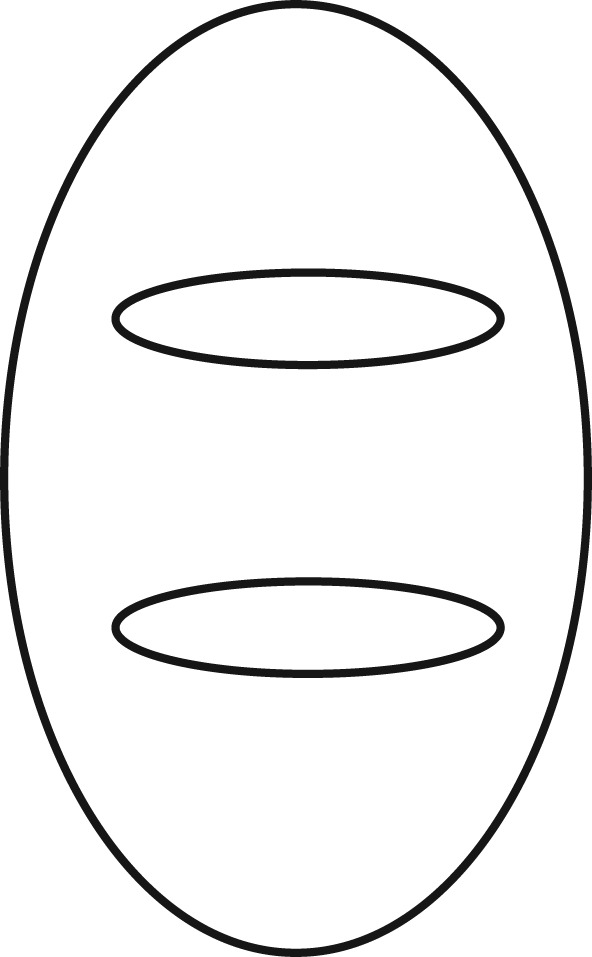
Droodle—what is it?

Amir *et al*. [19] attempted to find an fMRI signature for humour in an experiment that showed subjects simple ‘Droodle’ drawings [20–22] that could be simply described or given amusing interpretations (figure 1).

This is either:

Two smaller horizontal ellipses in a vertical ellipse, or

Closeup of a pig looking at book titles in a library.

This is an ingenious example of restricting inputs and thereby getting interpretable regularities in the output variables (from fMRI). Compare it with a parallel project (untried, to my knowledge!) to extract useful data from a variation on the *New Yorker*'s weekly contest to provide a funny caption for a cartoon. Subjects are all shown the same set of captionless drawings, and on some trials, subjects would be instructed to (try to) say something funny, and on other trials, simply to (try to) describe the picture. Even though different subjects would be given exactly the same visual stimuli on each trial, the variability of responses, both in the brain and in external behaviour, communicative and otherwise, would obviously defy statistical analysis. Thousands of entries are submitted every week [23] and probably in most cases those entries have been eventually chosen from a variety of candidates that ‘occur’ to the competitor, and then honed and honed and honed. Treating the first ‘output’ as indicative of the general power of human consciousness, rather than merely a semi-reliable marker of its instantaneous imbalance, would be myopic.

We know, anecdotally, that people's volatility of experience is a fact of life. Any attempt to measure this volatility experimentally is all but impossible, and so, the goal of explaining it scientifically is postponed or, more often, utterly ignored.

Another reason why the hard question is seldom asked is related to Richard Power's point about our lack of access to the properties of the medium of conscious experience. Think of the ‘miracle’ of vision. For millennia, reflective people from the pre-Socratic philosophers and Aristotle to the dawn of modern science with Descartes in the seventeenth century were quite baffled by the familiar fact that they could see things. Did something emanate *from* the eyes *to* the front surfaces of objects? What *entered* the eye, and where did it go once it was inside? Over the past few centuries, our understanding of how vision is accomplished has grown magnificently, and one of the striking facts about what we have learned is that until scientists told us, *we had no idea at all,* no ‘privileged access’, to the complicated activities of the optic nerve, the occipital cortex and even the activities of our eyeballs. Saccades were a surprising discovery to most people, as was the dramatically reduced acuity of perifoveal resolution, the absence of peripheral colour vision, the multiple ‘retinotopic maps’ in the cortex and much more. Science could inform us about all these amazing complexities, but *eventually,* it seemed, these processes yielded their results to wherever *we* were, our private, familiar world where we each, individually, *had access* to our subjective experiences. ‘Thanks, science, for laying the groundwork, but you are now entering a region where *we* are the authorities, and we'll take over the task of explanation, if you don't mind. Here is our phenomenological theory … derived by introspection’.

The problem is that this has seemed to make sense to too many researchers. They have acquiesced in the overconfident declarations of authority, which they probably found easy to endorse in their own cases. (For a vivid case of this assertion of authority, see Thomas Nagel's comment, and my response, at the NYU conference on animal consciousness, 17–18 November 2017 [24].) So they ceased, or postponed, or delegated the rest of the inquiry to the introspectors; their part of the job was complete: they had uncovered the unconscious and heretofore unimagined processes that deliver the goods ‘to consciousness’ and now it was somebody else's worry how to explain *that*. This is the setting, for example, in which some researchers conceive of their task as discovering the NCC, the neural correlates of consciousness, a process of systematic elimination until the necessary and sufficient conditions, the *causes*, of consciousness are pinned down in the brain while taking only sidelong glances at the typical *effects* of consciousness. This is an environment in which the theoretical illusion of Chalmers' hard problem can flourish.

## 3. Why the hard question is so hard

The fact is, the traditional claim that our conscious minds are immediately and maybe even perfectly known to each of us is wildly false. The psychologist Karl Lashley [25] once suggested provocatively that ‘no activity of the mind is ever conscious’, by which he meant to draw our attention to the inaccessibility of the *processing* that we know must go on when we think. What ‘we’ do ‘have access to’ is the *contents* and *apparent temporal order* of those contents, but how these contents, these representations of properties, objects and events, manage to represent what they do, and how they are generated when they ‘appear’ to ‘us’ is completely off-limits to introspection. Lashley gave an example: if asked to think a thought in dactylic hexameter, those who know which rhythm that is can readily oblige. For instance:

How in the world did this case of dactylic hexameter come to me?

We often know what we are trying to do, and why, because it is implicated by the context of the contents. When we hear a request to imagine a blue capital letter A against a black background, and accede, we are not surprised that we can do it, nor that we did it then, but we have no insight at all into how the ‘backstage’ neural machinery accomplished this. And when our minds are wandering, the succession of thoughts, images, melodies, pangs and thrills that occur to us arise from we know not where. How we do it, what goes on in us to produce such a thought, is something quite inaccessible to us. We can, however, reason plausibly about what *must* somehow be happening, beneath our notice. When we do this, we are not observing; we are theorizing. For instance, have you ever eaten a live frog? Have you ever strangled somebody to death with your bare hands? Your answers (I trust) are ‘instantaneous’, high-confidence and negative. How did you do it? You cannot tell by introspective observation; your convictions just ‘came to you’. But you can venture some canny theoretical hypotheses. Not by swiftly accessing your alphabetized and memorized list of Things I have never done (which has trillions of entries, of course). It must be—we reason—because we somehow (swiftly, without articulating it) recognize that *if* we had ever eaten a live frog or strangled somebody, we would not have forgotten it; it would now be flooding our conscious experience with garish details, and because nothing like that has arrived, we are quite sure it never happened. Contrast this with our *inability* to answer questions such as ‘Have you ever driven north for more than ten miles behind a Ford sedan?’ and ‘Have you ever had a day in the course of which you held a carving knife, two paperback novels, and a wool jacket?’ Our ability to answer some such questions and not others must depend, therefore, on our having a substantial self-knowledge that can somehow be tapped on the fly: ‘I'm the sort of person who would [not] forget that, if it ever happened’. Think how you would react to a new acquaintance who responded to the strangling question by mulling, scratching his chin and staring into the distance before answering!

Our self-knowledge is built up over a lifetime of experience, by processes we cannot observe, but we have learned that in many regards, we can trust it unreservedly. If it seems to us to be ‘knowledge by acquaintance’ and ‘immediate’, a special, well-nigh magical (or at any rate science-defying) kind of knowledge or authority, that is an illusion.

Another example of the sort of implicit and fallible reasoning that actually lies behind many of the overconfident declarations of person-first authority concerns the nature of colour experience, and more particularly, colour ‘qualia’. I have been denying the existence of qualia, as somehow directly apperceptible, intrinsic properties of conscious mental states for decades, and for all that time, many very thoughtful and well-informed people have scoffed dismissively. Imagine denying the existence of qualia! These wacky philosophers! No doubt, a good deal of the rejection of my position is due to my not having been able to replace their conception with a suitably persuasive alternative, but I can now say a little more—still not enough for some, I daresay—about what is there *instead of qualia*. (For an earlier attempt, see [26].) The imagination-stretcher I will use to present this alternative is owed, ironically, to Ned Block [27,28], one of the supreme defenders of qualia over the years.

Block argues for the existence of what he calls *mental paint*. The term, Block reminds us, was coined by Harman [29], and has been used by Block in defence of the view that there are properties of our conscious experience that are distinct from the properties (of things in the world) *represented* in our experience. In terms of Power's analogy, Block is insisting on *properties* of the medium, not the *content* of the medium at various times. Block ignores my derogatory term for the family of positions into which his falls. I spoke of views that posit *figment* [9]*.* Several cognitive scientists have confessed to me that they are strongly tempted to believe in figment, in spite of my arguments, and Block can be seen in his series of papers to be enthusiastically championing that view. Block also does not mention Zenon Pylyshyn's earlier disparagement of what he called ‘mental clay’ as a magical material out of which to fashion internal surrogates whose causal properties automatically track the physics of their external counterparts.

If magical mental clay is a bad idea, how about non-magical *virtual clay,* made of software, or information-processing? In virtual clay, you add only those properties you need for some purpose and simply leave out the rest. The difference between a real hotel and a virtual hotel is that in a real hotel, you have to devote lots of R&D, money and materials to make the rooms relatively soundproof, while in a virtual hotel, you have to devote lots of R&D and computer cycles and data structures to make the room *not* soundproof [30]. A virtual hotel room has within it only what you positively install, because it is something like an automated, dynamic *representation* of a hotel room.

One of my favourite examples is Donald Knuth's creation of *virtual glue* as an aid to creating the software typesetting system, TeX, one of the recognized masterpieces of software design. Virtual glue is a virtually elastic and virtually sticky substitute for the rigid space called up by the typewriter space bar. Putting a varying virtual dab between each pair of words, depending on their relative lengths, his formatting programme then virtually stretched the word-string or formula by virtually pulling on its virtual ends until it fitted perfectly between the left and right margins, apportioning just the right among of extra white space for each gap between words. TeX and its user-friendlier descendant LaTeX are the type-setting gold standard in scientific publication, where equations and other complex two-dimensional arrays of symbols are common, and now is used for many other page-design problems. Each word or symbol is put in a virtual box and, as one aficionado has put it,

Glue attaches boxes together: it defines how the boxes will be joined (should they line up their centers? Should they be aligned by some guideline?), and it defines how big the space between them should be, and how much it can be stretched or compressed. Page layout is really just tension relaxation: find the arrangement of boxes which produces the smallest overall tension, within the constraints imposed by the glue. [31]

Fortunately, Knuth did not also have to make his page-designing glue virtually shiny, tasty and smelly, but those are possible add-ons, if you have the time, the money and a good reason to do it. If we consider Block's mental paint to be *virtual* paint, his position becomes instantly more defensible, less of a fundamental challenge to cognitive science; it becomes a puzzle to be solved, not an impregnable mystery or an invitation to dualism. Block himself comes close to recognizing this:

I am not assuming that if there is mental paint, it is non-relational (intrinsic) or has no representational aspect. Since I favor physicalism, I allow that mental paint may be a relational neural property. To avoid misunderstanding: I do not claim that there is anything red or round in the head when one veridically sees a red or round thing in the world as when red pigment in a painting represents a red barn. ([28], fn2)

The virtue of virtual machines is that there is no point in imagining them unless you ask and answer the hard question. ‘OK, I'll give you virtual glue. And then what happens?’ Knuth's genius was seeing how he could create a dynamic data structure that could *do something,* could make a lot of hard work feasible by framing the problem in a certain way.

In general, one of the lessons of AI, or of computer science more generally, is that you never postulate a representation unless you postulate a representation-*user* whose ‘access to’ and ‘interpretation of’ the representation is definable, indeed implementable. This lesson has often been ignored in cognitive science and cognitive neuroscience, and for reasons that are not entirely disreputable. Almost everybody feels comfortable talking about *representation* in the nervous system these days, and about *information* being moved and processed and stored. The licence for this departure from the behaviourist strictures of yore is tacitly assumed to be that computer science has made it respectable to talk in these terms. And so it has, but until you can say just how (‘mechanistically’, in non-mentalistic, non-intentional terms) the information is interpreted, moved, stored and retrieved, you are taking out intelligence-loans that need to be repaid [32]. Deficit spending can be a useful, indeed well-nigh obligatory, policy when a scientific inquiry is in its infancy, as long as a path is left open to pay back the loans. (And if you do not pay off your loans, your project has an appropriately dismissive label: not software but *vaporware.*)

There could be no clearer example of this deficit spending than Block's postulation of mental (or virtual) paint. OK, so there is representation of something rather like paint (not like glue, not like sounds) that plays a role in visual perception, and we can confirm its existence (Block thinks) by considering empirical findings about measurable differences in subjects' performances in controlled experiments. The fact that Block pursues the strategy of discussing empirical experiments in detail shows that he is not falling in the typical philosophers’ trap of thinking the existence of what he is now calling mental paint is just obvious, a ‘direct’ deliverance of ‘first-person access’; he is accepting my challenge to *prove* that there are qualia, and in one sense that means that he is agreeing with me and *abandoning* qualia in one traditional sense: they are not being considered by him to be introspectible realities, readily confirmable by simply attending to one's experience. Mental paint, if it exists, is a posit of theory, a discovery of third-person science, not first-person observation.

This is a tempting idea precisely because it is *not* a claim about the (real) *properties* of the medium of representation, but about what the system of representation can *do,* thanks to its format. There is no second transduction into a mysterious medium, but there may well be a translation into a format that is user-friendly *from the point of view of the subpersonal consumers of the representations.* In other words, instead of qualia, we can have virtual paint; it just has to *do* whatever it is we think qualia do. What do we think they do? That is the hard question that must be asked. Otherwise, one is left marvelling at the mystery of it all.

The hard question here is definitely hard, because the brain's way of implementing virtual machines is not all that much like the well-understood ways that existing computers can implement virtual machines. The architectures are profoundly different, as are the components—networks of semi-autonomous, unsynchronized non-identical neurons, for instance, in contrast with the lockstep perfect-clone flipflops organized into battalions of memory registers with fixed addresses. The layers of computation that Knuth composed to implement virtual glue have no *obvious* counterpart in any neural states of excitation, so all we have at this point is a very promising hunch to follow up on, supported by an impressive existence proof: there exist virtual machines that can uncannily imitate real machines without any instantiation of the physical properties of those real machines.

## 4. Degrees of freedom and the control of consciousness

We have seen how we can partially explain the occurrence of some of the contents of our consciousness by relying on the contexts set up by the contents themselves: I am conscious of asking myself what I had for breakfast, and *presto!,* a memory of breakfast, with many unasked-for details, pops into my head. If I had not tried to recover this information, its arrival now might be troubling, or at least disconcerting, a case of ‘random’ mind-wandering of unknown significance. Why am I thinking about *this now*? And, we have seen how unnoticed and unnoticeable inferences (of sorts) ground some of our conscious convictions, but probably only a tiny subset of the transitions that occur in our ‘streams of consciousness' are due to anything as presentable as an inference. Most of the arrivals, unless we are working hard to pursue a line of thought, and succeeding, strike us as utterly out of our control. Moreover, we are deeply familiar with the experience of putting well-practiced activities such as driving or washing the dishes or mowing the lawn ‘on autopilot’ while letting our conscious minds wander pleasantly in the large space between daydreaming or fantasy, casual review of recent events, and barely intentional efforts at problem-solving. What governs all these transitions? Not a master scheduler, nor a Boss Neuron, nor a *homunculus* or *res cogitans.* It must be a dynamical, somewhat competitive process of contents vying for fame, for cerebral celebrity [4] or relative clout against the competition. What determines the winners? Something like micro-emotions, the strength of positive and negative valences that accompany, and control the destiny, of all contents, not just obviously emotionally salient events such as obsessive memories of suffering or embarrassment or lust, but the most esoteric and abstract theoretical reflections [33]. Marcel Kinsbourne has argued (M. Kinsbourne 2011, personal communication; see also [34]) that what makes *any* problem hard is that something false but attractive stands in its way. Solving hard problems takes more energy, more effort, more deliberate tactics of self-control, because tempting, easier, courses of action beckon. As I have often observed, *my* hard problem is finding more effective ways of showing people that Chalmers' hard problem is a chimera.

The general reason, I surmise, why thinking can be hard, and thinking about thinking can be even harder, is the omnipotentiality of content discussed in §2: the problem of control when there are millions of degrees of freedom. A hot-air balloon has only one readily controllable degree of freedom (up or down, by turning the heater on or off) out of which the experienced pilot can construct (from a very limited range of options) a desired trajectory through three-dimensional space by using knowledge of slight wind currents in relation to the terrain. Your vision has not just up–down, left–right, clockwise–counterclockwise (think of the pig reading the book title), but also vergence (close–far) and something like yaw (sideways motion of the head, to get parallax information about depth or distance). Any of these can be ‘clamped’, simplifying the control problem by removing one item that otherwise needs a ‘pilot’. When you succeed in concentrating on a problem, or just attending to your current task, you somehow block or damp down myriad alternative paths your mind could take you on. What informs and motivates the *you* that does this piloting? And how do you learn to be a reliable pilot? Again, not an inner homunculus, but a collaborative/competitive interaction between temporary coalitions of ‘interests’ [34]. And what emerges from this is the *virtual governor*. (Electrical engineers speak of a virtual governor as an emergent feature in a large system of generators linked together in parallel that maintains a more reliably constant frequency (e.g. 60 Hz) than any of the individual generators with their individual low-accuracy real, locatable governors. The virtual governor is not anywhere, but it seems to control the whole system [35].)

Every animal does an acceptable job of controlling its degrees of freedom under normal circumstances. Otherwise, it would be extinct. So, every human being does this as well, but in the human case, the task of governance is magnified by the essentially limitless numbers of degrees of freedom *that can iterate and ramify and amplify effects.* Taking care of this embarrassment of riches is—to oversimply—*what consciousness is for.* And its manner of action was succinctly expressed by Sarah McCarthy, the 4-year-old daughter of AI pioneer John McCarthy, when he asked her to do something. Her calm reply:

I can, but I won't.

Which, John McCarthy observed, was the core, the essence of free will (J. McCarthy 2005 and S. McCarthy, 2017, personal communication; see also [36]). The *conscious recognition* of (some of) one's many options combined with the competence to choose among them, for reasons good or bad, or for no reason at all, is the hallmark of responsible human agency. It is no mere coincidence that the philosophical problems of consciousness and free will are, together, the most intensely debated and (to some thinkers) ineluctably mysterious phenomena of all. As the author of five books on consciousness, two books on free will, and dozens of articles on both, I can attest to the generalization that you cannot explain consciousness without tackling free will, and vice versa. The key to a unified account lies, I now think, in the recognition (following Jablonka [37]) that the open-endedness of the human brain's representational power is, like the *unlimited heredity* [38] of evolution, both the problem and the solution to both mysteries. Free will, and consciousness, *matter* because we—and only we—must live in a world of our own creating that is orders of magnitude more complex and replete with *opportunities* (the degrees of freedom) than the lifeworld of any other living thing, and, with the help of evolution, both genetic and cultural, we have designed a system of higher-level cooperation that opens up modes of negotiation and mutually enforcible constraints, the civilization that makes life so worth living.

## Data accessibility

This article has no additional data.

## References

[1] Chalmers D 1995 Facing up to the hard problem of consciousness. J. Conscious. Stud. 2, 200–219.

[2] Nagel T 1974 What is it like to be a bat? Philos. Rev. 83, 435–450. (10.2307/2183914)

[3] Dennett D 1995 Facing backwards on the problem of consciousness. J. Conscious. Stud. 3, 4–6.

[4] Dennett D 1996 Consciousness: more like fame than television (in German translation) ‘Bewusstsein hat mehr mit Ruhm als mit Fernsehen zu tun’. In Die technik auf dem Weg zur seele (eds Maar C, Pöppel E, Christalle T), pp. 61–90. Berlin, Germany: Rowohlt.

[5] Dennett D 1998 The myth of double transduction. In Toward a science of consciousness II: The second Tucson discussions and debates (eds Hameroff S, Kaszniak AW, Scott AC), pp. 97–107. Cambridge, MA: MIT Press.

[6] Dennett D 2001 The Zombic Hunch: extinction of an intuition? In Philosophy at the new millenium (ed. O'Hear A), pp. 27 43. Cambridge, UK: Cambridge University Press.

[7] Dennett D 2002 Explaining the ‘magic’ of consciousness. In Proceedings of the International Symposium Exploring consciousness, humanities, natural science, Milan, Italy, 19–20 November 2001, pp. 47–58. Milan, Italy: Fondazione Carlo Erba.

[8] Dennett D 2005 Sweet dreams: philosophical obstacles to a science of consciousness. Cambridge, MA: MIT Press.

[9] Dennett D 1991 Consciousness explained. Boston, NY: Little Brown.

[10] Millikan RG 1984 Language, thought, and other biological categories: new foundations for realism. Cambridge, MA: MIT Press.

[11] Dennett D 1995 Darwin's dangerous idea: evolution and the meanings of life. New York, NY: Simon & Schuster.

[12] Kaufman S 2003 The adjacent possible. Edge.org, 9 November, https://edge.org/conversation/stuart_a_kauffman-the-adjacent-possible.

[13] McGinn C 1991 The problem of consciousness: essays toward a resolution. Oxford, UK: Blackwells.

[14] Povinelli D, Eddy TJ 1996 What young chimpanzees know about seeing. Monogr. Soc. Res. Child Dev. 61, 1–152. (10.2307/1166159) 8795292

[15] Call J, Tomasello M 2008 Does the chimpanzee have a theory of mind? 30 years later. Trends Cogn. Sci. 12, 187–192. (10.1016/j.tics.2008.02.010) 18424224

[16] Kaminski J, Call J, Tomasello M 2008 Chimpanzees know what others know, but not what they believe. Cognition 109, 224–234. (10.1016/j.cognition.2008.08.010) 18849023

[17] Dennett D 1983 Intentional systems in cognitive ethology: the ‘Panglossian Paradigm’ defended. Behav. Brain Sci. 6, 343–390. (10.1017/S0140525X00016393)

[18] Jackendoff R 2002 Foundations of language: brain, meaning, grammar, evolution. New York, NY: Oxford University Press. 10.1017/s0140525x03000153 15377127

[19] Amir O, Biederman I, Wang Z, Xu X 2013 Ha Ha! Versus Aha! A direct comparison of humor to nonhumorous insight for determining the neural correlates of mirth. Cereb. Cortex 25, 1405–1413. (10.1093/cercor/bht343) 24323497

[20] Price R 1976 Droodles #1. Los Angeles, CA: Price/Stern/Sloan Publishers Inc.

[21] Price R 2000 Droodles: the classic collection. Los Angeles, CA: Tallfellow.

[22] Price R 1955 Oodles of droodles. New York, NY: H. Wolff Book.

[23] Mankoff R 2010 How to win the cartoon caption contest. *New Yorker*, 6 October.

[24] T Nagel/DC Dennett 2017 exchange at NYU Conference on Animal Consciousness, 17–18 November 2017 (at 1 hour 23 minutes). See https://livestream.com/nyu-tv/AnimalConsciousness/videos/166146145.

[25] Lashley K 1958 Cerebral organization and behavior. Res. Publ. Assoc. Res. Nerv. Ment. Dis. 36, 1–18. 13527780

[26] Dennett D. 1994, Instead of Qualia. In Consciousness in philosophy and cognitive neuroscience (eds Revonsuo A, Kamppinen M), pp. 129–139. Hillsdale, NJ: Lawrence Erlbaum.

[27] Block N 2003 Mental paint. In Reflections and replies: essays on the philosophy of Tyler Burge (eds Hahn M, Ramberg B), pp. 165–200. Cambridge, MA: MIT Press.

[28] Block N 2010 Attention and mental paint. Philos. Issues 20, 23–63. (10.1111/j.1533-6077.2010.00177.x)

[29] Harman G 1990 The intrinsic quality of experience. Philos. Perspet. 4, 31–52. (10.2307/2214186)

[30] Dennett D 2001 Collision, detection, muselot, and scribble: some reflections on creativity. In Virtual music, computer synthesis of musical style (ed. Cope D), pp. 283–291. Cambridge, MA: MIT Press.

[31] Chu-Carroll M. (2008). http://scienceblogs.com/goodmath/2008/01/10/the-genius-of-donald-knuth-typ/.

[32] Dennett D 1971 Intentional systems. J. Philos. LXVIII, 87–106. (10.2307/2025382)

[33] Hurley M, Dennett D, Adams RB Jr 2011 Inside jokes: using humor to reverse-engineer the mind. Cambridge, MA: MIT Press.

[34] Ainslie G 2001 Breakdown of will. Cambridge, UK: Cambridge University Press.

[35] Hooker C 1989 Volutionary epistemology and the philosophy of science, part IV. In Issues in evolutionary epistemology (eds Hahlweg K, Hooker CA), pp. 119–121. Albany, NY: SUNY Press.

[36] McCarthy J 2002 Simple deterministic free will, unpublished memo, Published online, May 16, 2002, at www-formal.stanford.edu/jmc/freewill.html.

[37] Jablonka E 2017 Consciousness as we know it: the role of learning. In Presentation at NYU Conf. on Animal Consciousness, 17–18 November 2017 (at 11 minutes and 30 seconds). See https://livestream.com/nyu-tv/AnimalConsciousness/videos/166084404

[38] Szathmary E, Maynard Smith J 1995 The major transitions in evolution. Oxford, UK: Oxford University Press.

